# Does the severity of depressive symptoms after stroke affect long-term survival? An 18-year follow-up

**DOI:** 10.1371/journal.pone.0209157

**Published:** 2018-12-18

**Authors:** Mónika Kellermann, Roland Berecz, Dániel Bereczki

**Affiliations:** 1 Comedicum, Medizinisches Versorgungszentrum, München, Germany; 2 Department of Psychiatry, University of Debrecen, Debrecen, Hungary; 3 Department of Neurology, Semmelweis University, Budapest, Hungary; Chiba Daigaku, JAPAN

## Abstract

**Objective:**

We tested whether the severity of depressive symptoms in acute stroke and 4 years later are predictors of long-time survival.

**Method:**

We evaluated the severity of stroke in 82 patients with acute stroke by the Barthel index, the Scandinavian Stroke Scale and the Orgogozo scale, and we also quantified the severity of depressive symptoms by the Beck and the Hamilton scales in the first week of stroke, in 1995. We re-evaluated the scales 4 years after stroke in 41 out of 48 survivors. We checked the survival status of the initial cohort 18 years after stroke. In the assessment Kaplan-Meier graphs were constructed and the outcomes between groups were compared with log-rank tests.

**Results:**

Clinically important depressive symptoms (≥10 on the Beck scale) was present in 16 patients (19,5%) with acute stroke one week after admission. Case fatality was 41% at 4 years and 84% at 18 years after stroke. Those patients who survived at 4 years were significantly younger (p<0,05). Depressive symptoms in acute stage were not independent predictor of the length of survival. More severe strokes were associated with more severe depressive symptoms 4 years after stroke. In the survival subgroup of patients, those who had more severe depression (≥10 on the Beck scale) at 4 years, had shorter post-stroke survival than those with milder or no depression (Mann-Whitney test, p = 0.022; log-rank-test, p = 0.047). In multivariate analyses, adjusted for age, sex, stroke severity and the severity of depressive symptoms, age, sex and stroke severity remained the significant predictors of the length of survival.

**Conclusions:**

The severity of depressive symptoms either in the acute phase or 4 years after stroke is not an independent predictor of the length of survival in an 18-year follow-up.

## Introduction

Stroke represents the second most common cause of death worldwide following only coronary heart diseases [1], and it often has diverse, long-term physical and neuropsychological consequences [2]. Stroke is a leading cause of serious long-term disability in the United States [3]. Furthermore, WHO has projected that by 2030 unipolar depressive disorder will become the second leading cause of burden of disease worldwide, as measured by the disability-adjusted life years [4]. Several meta-analyses have confirmed a positive association between depression and the risk of cerebral ischaemic event [5,6].

Data available from 61 studies showed that depressive symptoms were present in 31% (28–35%) of all stroke survivors at any time during follow-up [7]. Ayerbe et al. [8] found similar estimates: the pooled prevalence of poststroke depression (PSD) at any time point was 29%, with a prevalence of 28% within a month of stroke, 31% at 1–6 months, 33% at 6 months to 1 year, and 25% at more than 1 year, respectively.

Depression after stroke is associated with a reduction in rehabilitation treatment efficacy [9, 10], limitations in daily life activities [11], and a higher risk of recurrent stroke [12]. Furthermore, poststroke depression was reported to be related to a considerably increased mortality risk [13], the odds ratio of mortality rate can be up 3,4 times in PSD patients compared to non-depressed post-stroke patients [8, 14]. The relationship between depression and mortality after stroke seems to be related to the follow-up duration [15]. Although the importance of poststroke psychiatric comorbidity is currently well documented, poststroke depression frequently remains undiagnosed [16].

Many different predictors of PSD were investigated across previous studies. Disability after stroke, cognitive impairment, stroke severity, history of pre-stroke depression, anxiety, and lack of family support were reported as predictors of depression after stroke in previous reviews [9,17]. Furthermore, the country of origin also seems to play a role [18]. The pathogenesis of PSD remains controversy whether PSD is a direct result of neuroanatomical impairment or indirectly due to the patient's abnormal reaction to a serious cerebrovascular incident [19]. However, long term data (15 years or more) on the relationship between depressive symptoms at the acute phase of stroke and the outcome of patients, measured by mortality, are scarce [20,21].

In a sample of 82 patients from 190 consecutive stroke patients admitted at our department in 1995, depressive symptoms were present in 22 patients (27%) one week after admission, and these patients had a significantly more severe stroke by Barthel, Scandinavian, and Orgogozo, scales (p<0,001) [22]. The aim of the present study was to analyse the presence and severity of depressive symptoms in stroke patients in acute stage as well as 4 years after stroke, and to test whether the severity of depressive symptoms is an independent predictor of case fatality during 18 years following the index stroke.

## Methods

### Examination in acute stage (</ = 7 days after stroke)

**Fig 1:**
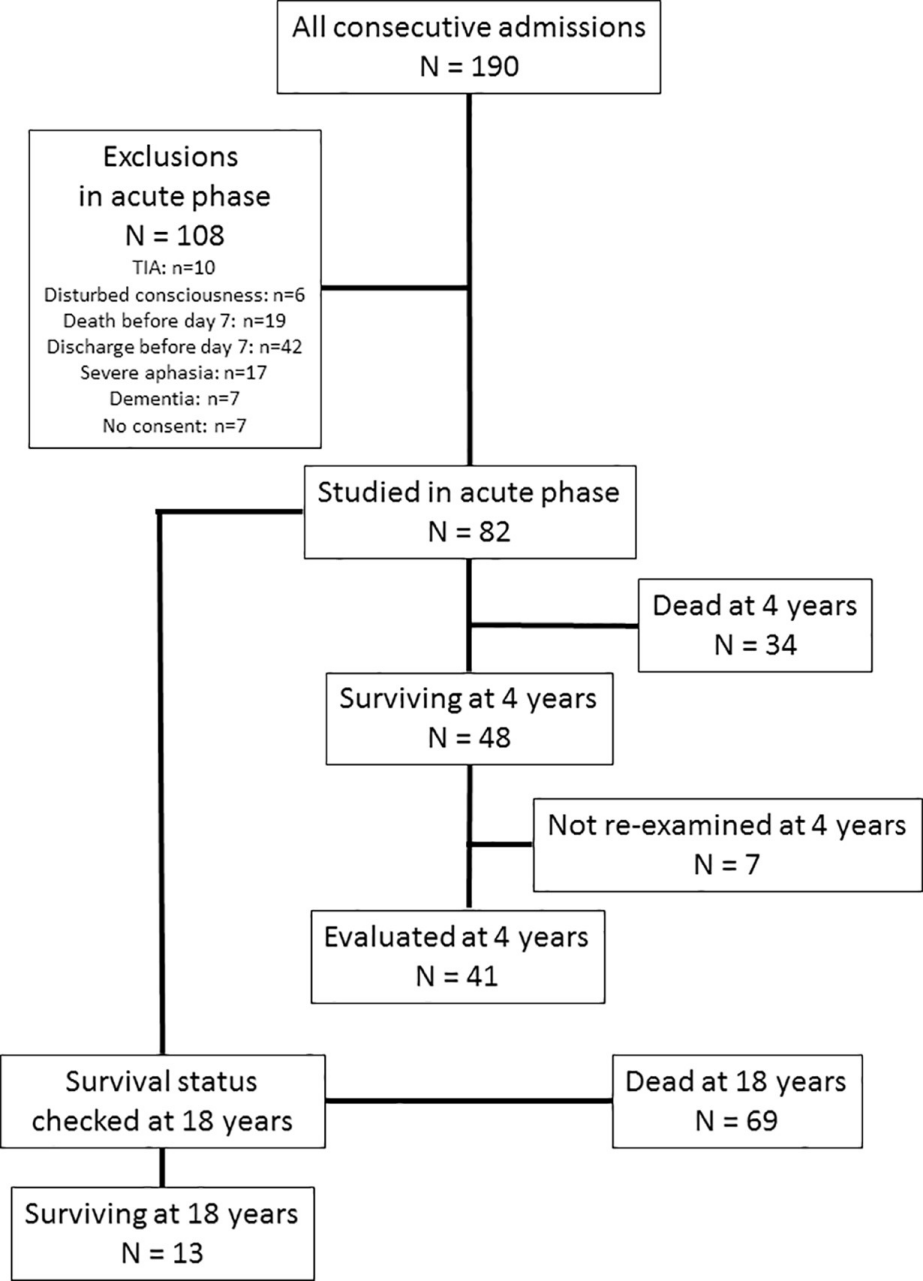
Patient flowchart.

During a 4-month period from September 1995 to January 1996, 190 patients with acute ischemic or hemorrhagic stroke were consecutively admitted to our stroke unit. Of these patients, 82 were eligible to be included in the study within one week after admission (Fig 1, for further details see [22]). Written informed consent was obtained from all 82 eligible patients. The study was approved by the local Ethical Committee of the University Medical School of Debrecen. To quantify the severity of stroke, we used the Scandinavian Neurological Stroke scale (SNSS, scoring range: 0–58, zero being the most severe) [23], the Orgogozo scale (ORG, scoring range 0–100, zero being the most severe) [24], and the Barthel index (BI, scoring range 0–100, zero being the most severe) [25] in the acute stage. The severity of depressive symptoms was evaluated by the 13 items version of the Beck Depression Inventory (BDI-13, scoring range 0–39, 39 being the most severe) [26] and the Hamilton Rating Scale for Depression (HRSD, scoring range 0–52, 52 being the most severe) [27]. The reliability and validity of these scale have been established previously [28–31]. Examination for the study was scheduled for the seventh day after admission, when the eligibility of the patients based on the exclusion and inclusion criteria were evaluated, and the stroke and depression scales were administered Exclusion criteria were transient ischemic attack; disturbance of the level of consciousness (somnolence, stupor, or coma), severe aphasia or dementia that prevented reliable evaluation of the mood with the applied scales. Patients who died or were discharged before the planned day of examination were also not included. Details of our findings in the acute stage have been previously reported [22].

### Follow-up examinations (4 and 18 years after stroke)

At four years after stroke, the same investigator (M.K.) personally visited and re-evaluated each patient at his or her home. Four patients could not be traced at their prior address, two patients refused to follow-up, while one patient was unable to adequately cooperate due to severe aphasia. The BI, the SNSS, the ORG and the BDI-13 scores could be evaluated in 41 out of 48 survivors at four years after stroke. Survival status and date of death were checked from the database of the National Health Insurance Fund at 18 years after stroke (Fig 1).

We used the Spearman correlation to evaluate the association between initial stroke severity, age and the severity of depressive symptoms 4 years after stroke. Initial and 4-year values for scale scores were compared by the Wilcoxon matched pairs test, and the association of initial and 4-year score values were evaluated by the Spearman correlation coefficient. Initial age, stroke and depressive symptoms severity of survivors at 4 years and deceased patients were compared by parametric t-test (in case of age) and non-parametric Mann-Whitney test (for scales). Kaplan-Meier curves were constructed, and the log-rank tests were used to compare the survival distributions between patient groups using 2 cutoff values (5 and 10) for the 13-item version of the BDI-13 (where higher scores mean more severe depression) [32]. In multivariate testing by General Lineal Model (GLM), age at stroke onset, sex, a measure of stroke severity (SNSS) and BDI-13 score were entered in the model to identify independent predictors of the length of survival (measured by years). Statistical significance was set at p <0.05. Statistica for Windows v. 11 (StatSoft, Tulsa, OK) was used for data analysis.

## Results

**Fig 2:**
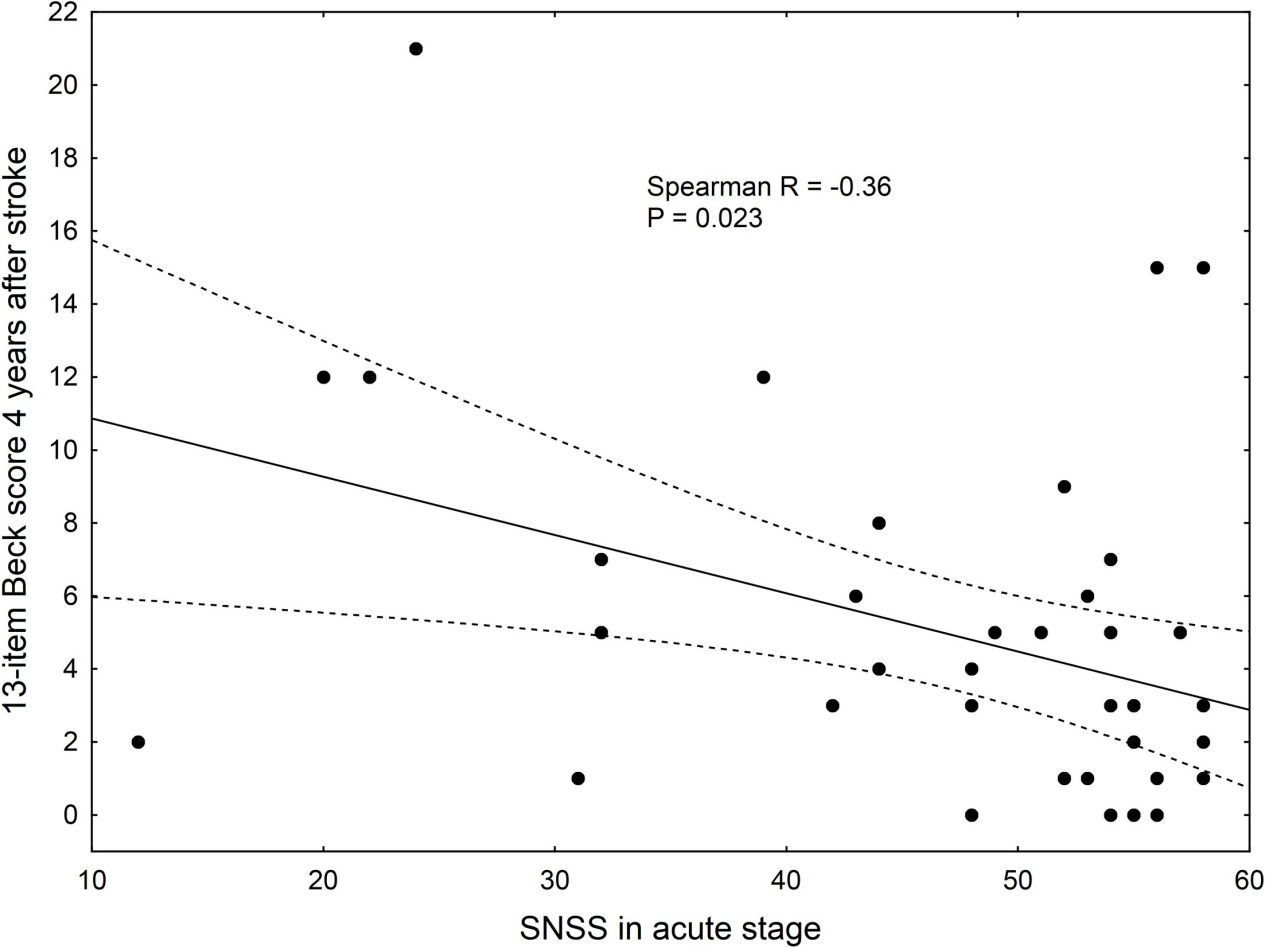
More severe signs of stroke in the acute stage are associated with more severe depressive symptoms 4 years after stroke. The dotted lines denote 95% confidence interval around the regression line. SNSS: Scandinavian Neurological Stroke Scale score (higher scores mean less stroke severity).

In the acute stage, 82 patients with acute stroke were included in this study. There were 48 survivors (59%) at 4 years and 13 survivors (16%) at 18 years after stroke. Those patients who survived at 4-years were significantly younger (63,0±13,9 versus 70,0±9,4, p<0,05). The severity of stroke signs and depressive symptoms in those patients surviving at 4 years are presented in Table 1. At 4 years after stroke, scores on the stroke scales as well as on depression scales correlated with both stroke and depressive scores in the acute phase. We found that more severe strokes were associated with more severe depressive symptoms 4 years after stroke (Fig 2). Those who had a moderate or severe depression (scored ≥10 on the Beck scale) at 4 years had a shorter post-stroke survival than those scoring below 10 (7.2±1.9 vs 12.7±5.1 years, p = 0.022).

**Table 1:** The severity of stroke signs and depressive symptoms in the acute phase, and in survivors 4 years after stroke (n = 41).

Feature	In the 1^st^ week after stroke		At 4 years after stroke (n = 41)	P (Wilcoxon matched pairs tests)	Spearman R (p)
	Total initial sample (n = 82)	Initial value in those who will survive at 4 years (n = 41)			
BI	65 ± 34	72 ± 33	86 ± 24	0.002	0.55 (<0.001)
BDI-13	4.4 ± 4.7	4.2 ± 4.9	4.5 ± 4.7	0.7	0.47 (0.002)
HRDS	5.4 ± 6.9	5.2 ± 6.7	9.2 ± 7.8	0.004	0.44 (0.009)
ORG	76 ± 24	79 ± 23	87 ± 18	0.002	0.64 (<0.001)
SNSS	45 ± 12	47 ± 12	52 ± 9	<0.001	0.69 (<0.001)

Values are mean ± SD

BI = Barthel Index, BDI-13 = Beck Depression Inventory, HRDS = Hamilton Rating Scale for Depression, ORG = Orgogozo scale, SNSS = Scandinavian Neurological Stroke scale

Initial stroke severity and the severity of depressive symptoms at 4 years but not in the acute stage were associated with the length of survival after stroke (Spearman R, p<0.05, Table 2).

**Table 2:** Predictors of length of survival.

Predictor	Spearman R (p)	P by multivariate testing
Age	-0.35 (0.001)	< 0.001
Sex	n/a	0.03
Acute stroke severity (n = 82)		
BI	0.31 (0.005)	
ORG	0.23 (0.043)	
SNSS	0.26 (0.020)	0.005
Acute symptoms of depression (n = 82)		
BDI-13	-0.02 (0.8)	0.41
HRDS	-0.04 (0.7)	
Stroke severity at 4 years (n = 41)		
Barthel Index	0.42 (0.007)	
Orgogozo	0.35 (0.025)	
SNSS	0.43 (0.006)	0.059
Symptoms of depression at 4 years (n = 41)		
BDI-13	-0.32 (0.04)	0.12
HRDS	-0.33 (0.04)	

BI = Barthel index, ORG = Orgogozo scale, SNSS = Scandinavian Neurological Stroke scale, BDI-13 = Beck Depression Inventory, HRDS = Hamilton Rating Scale for Depression.

**Fig 3:**
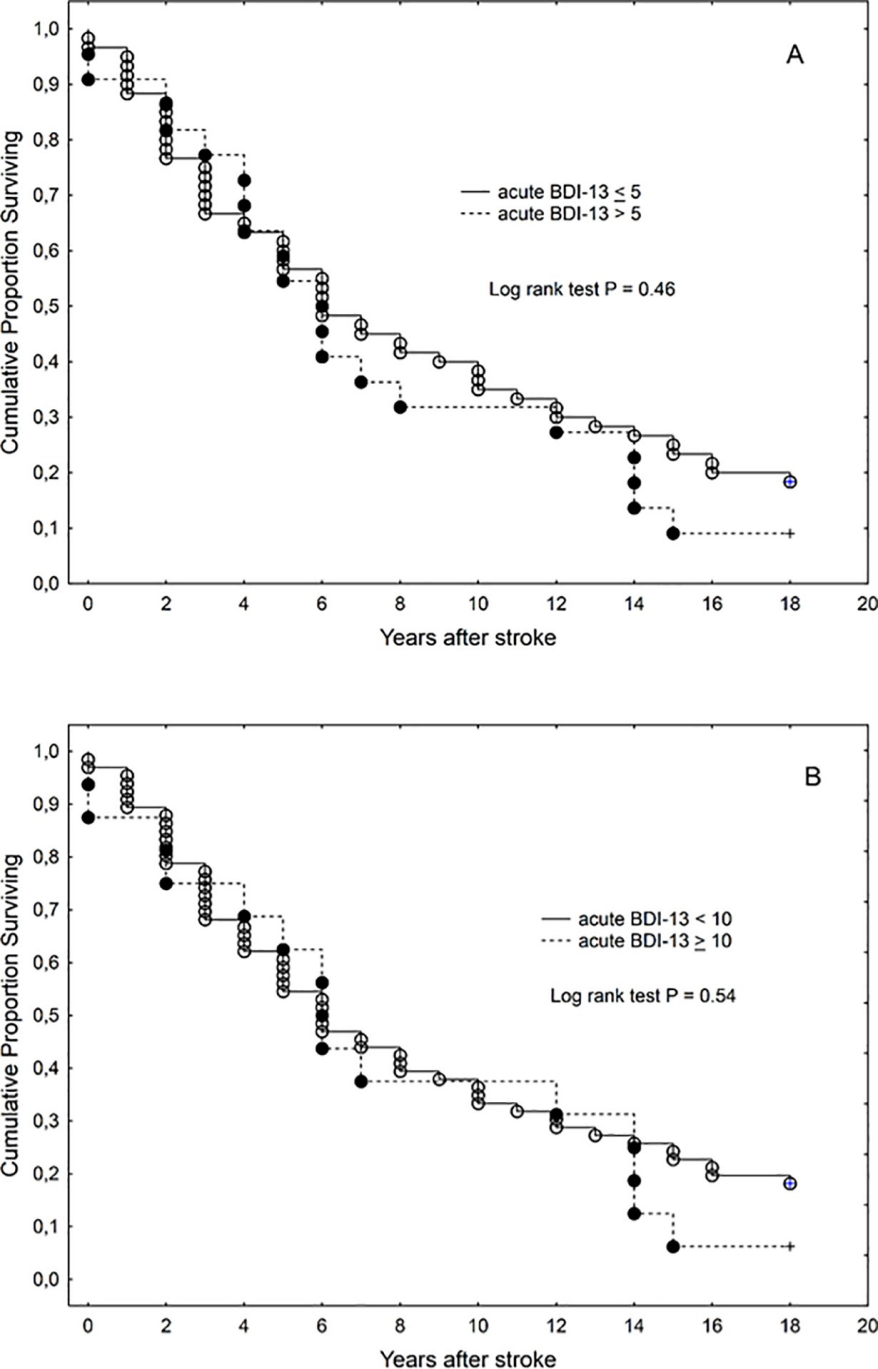
**Kaplan-Meier graph of survival in those with Beck Depression Inventory score cutoff values of 5 (A) and 10 (B) in the acute stage (n = 82)**. BDI-13 = Beck Depression Inventory.

**Fig 4:**
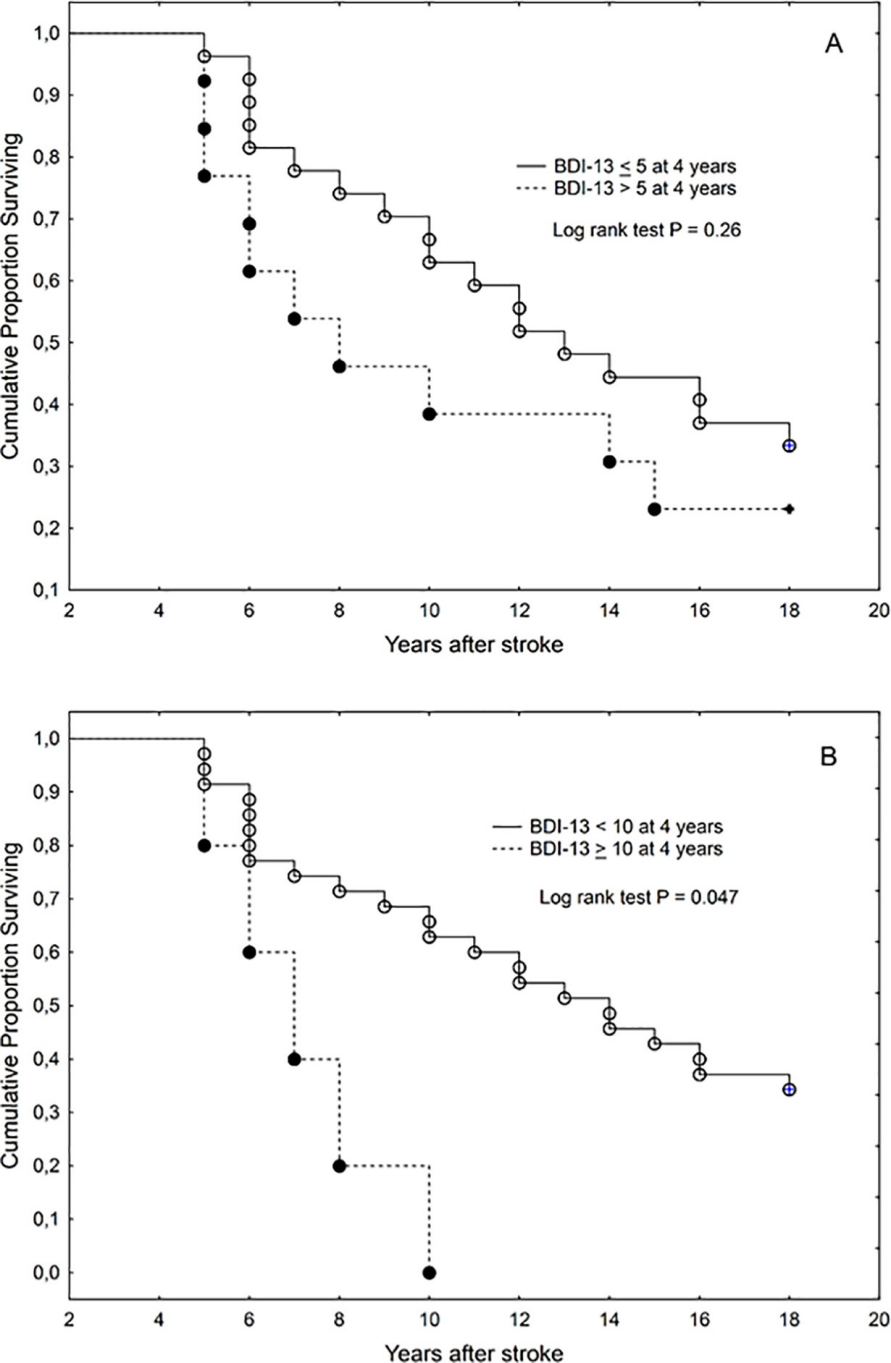
**Kaplan-Meier graph of survival in those with Beck Depression Inventory score cutoff values of 5 (A) and 10 (B) at 4 years after stroke (patient subgroup survived at 4 years, n = 41)**. BDI-13 = Beck Depression Inventory.

Although Kaplan-Meier curves suggested shorter survival in those with more severe depressive symptoms (using cutoff values 5 and 10) in the acute stage as well as at 4 years (Figs 3 and 4), in multivariate analyses only age, sex and stroke severity remained significant independent predictors of the length of survival

## Discussion

To our knowledge, only few studies investigated the predictive value of depressive symptoms more than 15 years after stroke [20,21]. In the present study, we found that more severe strokes are associated with higher Beck scores at 4 years after stroke. However, the severity of depressive symptoms either in the acute phase or 4 years after stroke is not an independent predictor of the length of survival. Due to the strong effect of stroke severity and age on survival, there was only a trend towards an association between the severity of depressive symptoms after stroke and case fatality.

The predictors of poststroke depression have been studied in the past few decades. Similar to the results of De Ryck [33] and Hermann [34] we found a correlation between stroke severity and depressive symptoms. Systematic reviews on this topic also found that stroke severity is consistently associated with higher rates of depression after stroke [8,17].

A systematic review and meta-analysis of studies found increased/ early mortality at follow-up for individuals with PSD [8,15]. Based on a meta-analysis be Bartoli et al. [15] the relationship between depression and mortality after stroke is related to the follow-up duration. The short-term studies (<2 years) did not show a statistically significant association between depression after stroke and mortality; on the other hand, in medium term (2–5 years) follow-ups the association was statistically significant and the analysis of long-term investigations (>5 years) showed some trend [15]. The results of our study showed that the patients who survived at four years were significantly younger and the initial age at the time of the acute stroke was the main predictor of survival. However, in the subgroup of the initial patients’ population who survived at 4 years the severity of depression correlated with the length of survival.

Depression may affect the prognosis and risk of mortality after stroke because stroke patients suffering from depression may be less compliant to treatment [35]. Depression is associated with poor health behaviours (i.e. smoking, physical inactivity, poor diet, lack of medication compliance) which have a negative impact on survival [36]. Furthermore, depression is related to other major comorbidities, such as diabetes, hypertension, neuroendocrine changes (e.g., sympathetic nervous system activation, dysregulation of the hypothalamic-pituitary-adrenocortical axis), platelet aggregation dysfunction [37], and immunological, inflammatory changes [38].

Several randomized, double-blind, controlled studies have examined the efficacy of pharmacotherapies in PSD. In a network meta-analysis paroxetine was found to be the most effective, followed by imipramine, reboxetine, nortriptyline, citalopram and fluoxetine [39].

Jorge et al. [40] could show that treatment with antidepressants, fluoxetine or nortriptyline, for 12 weeks during the first 6 months after stroke significantly increased the survival of both depressed and non-depressed patients. Additionally, a multicenter randomised controlled trial found, that citalopram reduced disability and cardiovascular mortality following stroke [41]. in a meta-analysis published by Salter et al. [42], the results showed that the early initiation of antidepressant therapy in non-depressed stroke patients might reduce the odds for development of poststroke depression. Finally, problem-solving therapy for 1 year after the stroke increased the time to mortality in a long-term follow up [43].

These findings suggest that the pathophysiological processes determining the increased mortality risk might be associated with poststroke depression.

A major limitation of this study is the relatively small sample size of our patient group, however, the prospective nature of the study, and the use of standardized scales for the evaluation of both stroke and depression severity increase the reliability of the data. The results of the study is also strengthened by the repeated evaluation of the patients 4 years after stroke, as well as the uniquely long duration with a low (<3%) rate of loss to follow-up regarding survival data. Another limitation was the relatively high number of patients who were excluded from the study in the acute phase. Those patients, who had a severe stroke and died before the planned day of examination were not included, similarly those who had mild stroke symptoms and were discharged before day 7. The study therefore excluded the most severe and milder cases (for detailed analysis see [22]). However, the tested population was therefore more homogenous and most relevant for the long-term evaluation of the outcome status after stroke. At 4-year follow-up seven patients either could not be traced at their permanent or temporary address or refused to consent to the follow-up. We cannot exclude that these patients might have different characteristics compared to the tested population regarding their depression status.

Further research is needed to clarify the association between poststroke depression, mortality after stroke and related pathophysiological processes. Secondly, the effectiveness of pharmacotherapy and psychotherapy for preventing and treating poststroke depression should be explored.

## Conclusion

In an 18-year follow-up of an initial cohort of acute stroke patients, we found that more severe strokes were associated with more severe depressive symptoms 4 years after stroke. Those who had more severe depressive symptoms at 4 years had a shorter post-stroke survival. In multivariate analyses only age, sex and stroke severity remained significant predictors of the length of survival. The severity of depressive symptoms either in the acute phase or 4 years after stroke may not be an independent factor of the length of survival in an 18-year follow-up.

## Supporting information

S1 Table Individual patient data in xls format. (XLS)
